# Severe facial necrosis in a type 1 diabetic patient secondary to mucormycosis masquerading as an internal maxillary artery occlusion: a case report

**DOI:** 10.1186/s12879-019-3822-9

**Published:** 2019-02-22

**Authors:** Farheen Manji, John C. Lam, Bonnie L. Meatherall, Deirdre Church, Bayan Missaghi

**Affiliations:** 1 Alberta Health Services, Calgary, Canada; 2 University of Calgary, Calgary, Canada; 3 Calgary Laboratory Services, Calgary, Canada

**Keywords:** Mucormycosis, Internal maxillary artery occlusion, Rhinosinusitis

## Abstract

**Background:**

Mucormycosis is a group of rare but life threatening angioinvasive infections caused by fungi of the order *Mucorales* that often occurs in immunocompromised patients and individuals with poorly controlled diabetes. Rhinocerebral mucormycosis can mimic sinusitis but can rapidly progress to deeper disease and cause facial necrosis. Facial vascular thrombosis is a rare complication of mucormycosis and can confound diagnosis of the disease.

**Case presentation:**

We report the case of a 25-year-old female with poorly controlled type 1 diabetes mellitus who initially presented with symptoms of sinusitis but rapidly progressed with signs of left-sided facial necrosis due to occlusion of the left internal maxillary artery. Early surgical debridement did not yield a microbiological diagnosis. Deeper surgical debridements ultimately revealed angioinvasive fungal disease consistent with mucormycosis. The patient recovered after repeated surgical intervention and aggressive parenteral antifungal therapy.

**Conclusion:**

This case illustrates an atypical complication of mucormycosis, and emphasizes that a high index of suspicion in vulnerable patient populations aids in the diagnosis of this life-threatening infection.

## Background

Mucormycosis is a group of rapidly progressive angioinvasive fungal infections with significant morbidity and mortality [1]. We present the case of a 25-year-old female with poorly controlled type 1 diabetes mellitus presenting with rapidly progressive left-sided facial necrosis due to occlusion of the left internal maxillary artery that was ultimately discovered to be secondary to invasive mucormycosis.

## Case presentation

A 25-year-old female with poorly controlled type 1 diabetes mellitus presented to hospital for the second time in two weeks with recurrent, antibiotic-refractory left sided facial swelling and pain complicated by diabetic ketoacidosis (DKA). There was no history of antecedent dental manipulation. Two weeks prior, she was seen in an ambulatory clinic for the same symptoms and took a three-day course of amoxicillin-clavulanic acid 875/125 mg twice daily but was admitted to hospital three days later for DKA. During this index hospitalization, her diagnosis was correlated radiographically and presumed to be sinusitis complicated by DKA. A two-day course of ceftriaxone 2 g intravenously once daily and vancomycin 1 g intravenously twice daily was administered before transitioning to doxycycline 100 mg twice daily for an additional ten-day course.

She returned to hospital a mere ten days later with progressive left-sided facial swelling and was found to meet biochemical criteria for DKA. She was afebrile and hemodynamically stable but had profound left periorbital edema with necrotic lesions along her left maxillary region and forehead.

Three sets of blood cultures, each consisting of an aerobic and anaerobic bottle pair (20 mL per bottle), were drawn before any further parenteral antibiotics were given remained negative after 4-days of incubation in a BacT-Alert automated system (bioMérieux, Laval, Quebec). HIV serology was negative. Comparison of a repeat computed tomography (CT) scan of her sinuses with CT images performed during her prior hospitalization demonstrated improved aeration of the left maxillary sinus but progressive left facial soft tissue swelling complicated by subcutaneous emphysema. Subsequent CT-angiogram of the neck revealed an internal maxillary artery occlusion.

Although initial nasal rhinoscopy revealed normal appearing sinus tissue, surgical debridement to the epicranial aponeurosis revealed necrotic tissue with poor vascular supply but no microbiological diagnosis.

**Fig. 1:**
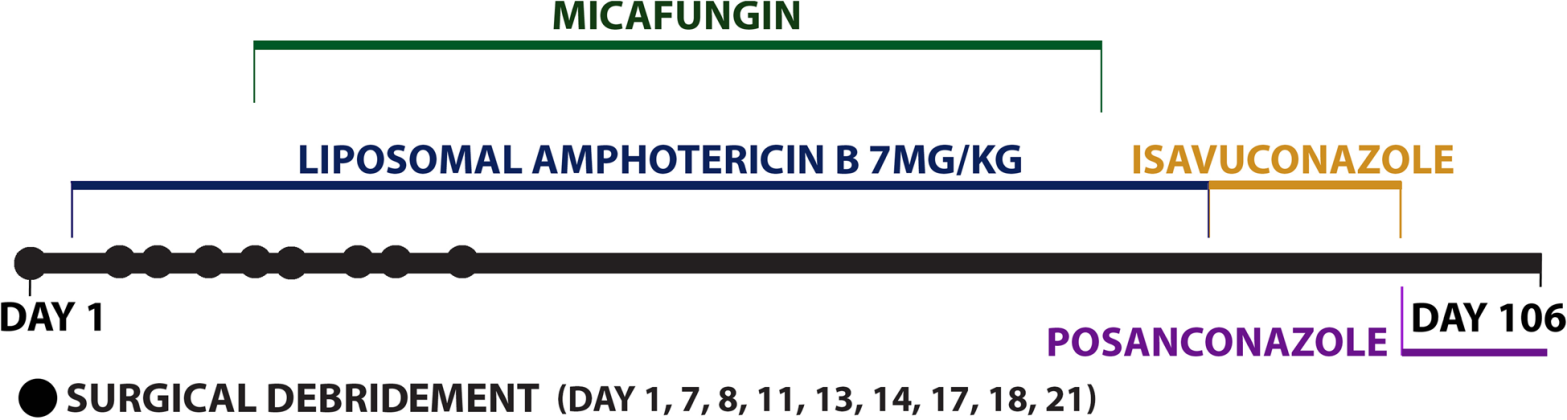
Timeline of events including the patient’s surgical debridements and antifungal therapy over the course of her 106 day stay in hospital

She was started empirically on parenteral therapies of piperacillin-tazobactam 3.375 g every six hours, vancomycin 1250 mg every twelve hours and liposomal amphotericin B at 7.5 mg/kg once daily before transfer to a tertiary care center for further surgical consultation. The ischemic changes on her face was originally thought to be secondary to the internal maxillary artery occlusion from surrounding inflamed tissue. Given the extent of her rhino-orbital disease, she underwent surgical debridements. Her timeline is outlined in Fig. 1.

**Fig. 2:**
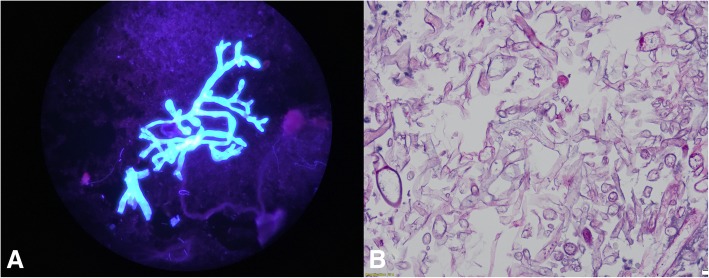
**a** Calcofluor white stain from deep buccal biopsy demonstrating wide pauci-septated branching, 100x magnification (**b**) Periodic Acid Schiff stain from periorbital biopsy illustrating numerous pauci-septated ribbon like fungal organisms with occasional right-angle branching

Original tissue cultures, including anaerobic, fungal and acid-fast bacilli cultures set-up under various growth, differential and selective media for pathogen identification were negative. Four subsequent deeper debridements were done, which extended down to the sternocleidomastoid muscle and up to the auditory canal and as deep as the skull base. Histopathology from biopsied tissue and bone from these sites revealed pauci-septated, ribbon shaped elements with liquefactive skin necrosis consistent with mucormycosis (Fig. 2 a and b). Microbiological 18 s RNA sequencing ultimately identified *Rhizopus oryzae* as the causative organism. Her hospitalization was complicated by kidney injury secondary to hypovolemia and amphotericin B, necessitating intermittent hemodialysis but she subsequently recovered her renal function. Due to disfiguration from extensive debridement, she underwent a facial skin graft reconstruction after achieving surgical care of her invasive fungal infection. She was transitioned to isavuconazole for three months and then to posaconazole which she continues indefinitely.

## Discussion and conclusions

Mucormycosis is an angioinvasive infection caused by fungi of the order *Mucorales. Mucorales* are ubiquitous in the environment and cause invasive disease in immunocompromised patients, particularly those with poorly controlled diabetes, hematological malignancy, post-transplant status, and iron overload states. [1]. *Rhizopus* is the commonest isolated agent of mucormycosis in humans [2].

Mucormycoses traditionally present as rhinocerebral disease but can also manifest as pulmonary, cutaneous, or gastrointestinal diseases. [3]. Inhalation of environmental fungal spores seed the sinuses and progress, leading to facial pain, swelling or numbness with possible deeper extension involving the orbits and brain. Complications such as vision loss, cerebral abscesses and hematologic dissemination with organ seeding have been documented [2].

The absence of a pathological diagnosis on index rhinoscopy was initially falsely reassuring as a microbiological diagnosis was secured only after deeper debridements were undertaken. While there are case reports describing carotid artery, cavernous sinus and internal jugular vein involvement with mucormycosis, there are only three case reports in the literature describing an internal maxillary artery occlusion [4–6]. Our case was further confounded by absence of pathology on rhinoscopy.

The mainstay of mucormycosis treatment lies in aggressive surgical source control with adjunctive intravenous antifungal therapy and management of predisposing factors [3]. Intravenous liposomal amphotericin B is used for invasive mucormycosis given its ability to penetrate the central nervous system [3]. Salvage therapies, including posaconazole and isavuconazole, have promising but limited evidence and ought not be considered first line medical therapy [7–9].

The case described herein illustrates the need to maintain a high index of suspicion for invasive mucormycosis in a high-risk host with a compatible syndrome. Recognition of vascular thrombosis as a complication secondary to angioinvasive dissemination rather than a primary cause of necrosis must be considered. As such, repeat biopsies may be necessary in order to identify mucormycosis – which is particularly important given its high mortality. Histopathology is the gold standard for diagnosis but should not preclude early empiric antifungal therapy.

## Abbreviations

CT: Computed tomography
DKA: Diabetic ketoacidosis
